# Functional validation of *CHMP7* as an ADHD risk gene

**DOI:** 10.1038/s41398-020-01077-w

**Published:** 2020-11-06

**Authors:** Callum Dark, Caitlin Williams, Mark A. Bellgrove, Ziarih Hawi, Robert J. Bryson-Richardson

**Affiliations:** 1 School of Biological Sciences, Faculty of Science, Monash University, Clayton, Australia; 2 Turner Institute for Brain and Mental Health, School of Psychological Sciences, Monash University, Clayton, Australia

**Keywords:** ADHD, Medical genetics

## Abstract

Attention deficit hyperactivity disorder (ADHD) is a neurodevelopmental disorder of childhood with a strong genetic component. Despite the success of mapping ADHD risk loci, little work has been done to experimentally verify the contribution of these loci to ADHD phenotypes. Meta-analysis of four genome-wide association studies in ADHD suggested *CHMP7* as a predisposing gene for ADHD. A DNA variant (rs2294123) mapped to *CHMP7* has been shown (via bioinformatic analysis) to have a high likelihood for functionality and correlate with reduced transcript levels. We used CRISPR-Cas9 genome editing to generate a *chmp7* zebrafish model for ADHD. *chmp7*^*+/−*^ fish showed comparable reductions in mRNA levels to individuals homozygous for the *CHMP7* ADHD risk allele. These fish displayed significant hyperactivity over a 24-h period at 6 days post-fertilisation compared to *chmp7*^*+/+*^, but this effect did not persist into juvenile and adulthood stages. In addition, *chmp7*^*+/−*^ fish had significantly smaller total brain volumes than *chmp7*^*+/+*^ fish. Finally, the hyperactivity at 6 days post-fertilisation was significantly reduced through the application of methylphenidate, a mainstay pharmacological treatment for ADHD. Overall, this study highlights an important role for *CHMP7* in the neurodevelopment of ADHD, and demonstrates the utility of zebrafish for modelling the functional effects of genes conferring risk to ADHD.

## Introduction

Attention deficit hyperactivity disorder (ADHD) is a highly prevalent neurodevelopmental disorder that affects ~5% of school age children^1^ and persists into adulthood in 30–60% of cases^2,3^. Reductions in brain volume are also often associated with the disorder^4^.

It is estimated that genetic factors account for ~80% of ADHD’s aetiology^5,6^. Meta-analyses of genome wide association studies (GWAS) identified several DNA variants potentially involved in ADHD, including rs2294123, which maps to charged multivesicular body protein 7 (*CHMP7*^7^). In addition, recent large scale GWAS^8^ demonstrates that rs2294123 is in significant LD with several SNPs showing nominal association with ADHD (Supplementary Fig. 1, Supplementary Table 1). However, functional validation of the contribution of these associated genes to the development of ADHD is lacking. Most variants detected via candidate gene and GWAS approaches map to non-coding regions of the genome^9^. Given the wide range of roles that non-coding regions play in gene expression, separating neutral ADHD-associated variants from causative/pathogenic ones remains a major challenge.

To tackle this, Tong et al.^9^ developed an in silico pipeline to prioritise a curated set of non-coding single nucleotide polymorphisms (SNPs) linked to ADHD for functional analysis. They identified an SNP (rs2294123, G→T) mapped to the 5ˈUTR of *CHMP7*, which was significantly associated with ADHD. Homozygosity for the risk allele (T) was significantly associated with lower neurocognitive function in ADHD individuals, higher ADHD symptom traits, and a 33% reduction in *CHMP7* transcript levels compared to homozygous G individuals^9^. These findings suggest that a reduction in *CHMP7* expression contributes to ADHD phenotypes and warrants further investigation.

CHMP7 plays an important role in endosomal sorting^10^, nuclear envelope formation^11^, and has recently been implicated in spinal and bulbar muscular atrophy^12^. It interacts with a member of the endosomal sorting complex required for transport (ESCRT) family, ESCRT-III^10^. Several CHMP family members are part of ESCRT-III, have been implicated in neurodevelopmental processes^13^, and are known to contribute to the development of neuropsychiatric conditions^14–16^. This supports the need for further examination of the role of *CHMP7* in ADHD.

To examine the functional relevance of *CHMP7* to ADHD, we generated a *chmp7* zebrafish mutant line using CRISPR-Cas9 genome editing, and hypothesised that *chmp7* heterozygous (*chmp7*^*+/−*^) animals would mimic the reduction in transcripts correlated with the ADHD-associated SNP (rs2294123^9^). We demonstrate that *chmp7*^*+/*−^ fish are more active than wildtype (*chmp7*^*+/+*^) and have decreased total brain volumes, similar to that reported in ADHD cases. Furthermore, we also show that hyperactivity of *chmp7*^*+/*−^ fish can be significantly reduced through the application of methylphenidate, which is commonly used for the treatment of ADHD. Overall, we have provided experimental validation for *CHMP7* as a risk factor for ADHD.

## Materials and methods

### Ethics

Fish were maintained in the Monash University Fish Core facility under breeding colony license MARP/2015/004/BC. Creation of transgenic lines was approved by the School of Biological Sciences Animal Ethics Committee (BSCI/2015/07). Experiments were carried out on embryos of wildtype (*Tübingen*, TU) background.

### Generation and genotyping of the *chmp7* mutant line

To create a *chmp7* mutant zebrafish line, a guide RNA targeting *chmp7* (ENSDARG00000041362) exon 2 was generated according to Gagnon et al.^17^. One-cell stage embryos were injected with a mixture containing: 150 ng/µL guide RNA, 5 µg/µL Cas9 protein (PNA Bio, Newbury Park, CA, USA), 20 µM STOP cassette, 0.25 µL Phenol Red, 0.25 µL Cascade Blue (Molecular Probes, Waltham, MA, USA), and ultra-pure H_2_O to a final volume of 2.5 µL. Embryos were selected for Cascade Blue, indicating successful injection, at 24 hpf and raised to adulthood. F_0_ founders were identified by outcrossing to TU fish, collecting DNA from 15 to 20 offspring, and the pooled DNA used as a template for PCR amplification of the region surrounding the target site. Polyacrylamide gel electrophoresis was used to visualise heterodimers. Founders were outcrossed to wildtype fish, and F_1_ individuals screened for the presence of mutations using PCR and gel electrophoresis. The mutation was determined using Sanger sequencing. Experiments utilised F_3_ and subsequent generations. Guide RNAs and primer sequences are presented in Supplementary Table 2. Genotyping was performed using allele-specific KASP fluorescence assays (LGC Biosearch Technologies, Teddington, Middlesex, UK).

### Whole-mount in situ hybridisation

To generate a probe to examine *chmp7* expression, a *chmp7* fragment was amplified from genomic DNA using primers (Supplementary Table 2) and cloned into pGEM-T Easy (Promega, Madison, WI, USA). Sequence orientation was determined via PCR using combinations of *chmp7* and M13 primers (Supplementary Table 2). Probe templates were amplified from the plasmid using the *chmp7* in situ template reverse and pGEM-T Easy M13 forward primers, and digoxygenin riboprobes generated using T7 RNA polymerase as previously described^18^. Whole-mount in situ hybridisation was performed as per Ruparelia et al.^19^.

### Reverse transcription-PCR

RT-PCR was utilised to determine when *chmp7* is expressed. cDNA synthesis was performed on RNA extracted from wildtype embryos at the 8- and 16-somite stages, 1, 1.5, 2, 3, 4, and 5 dpf. Total RNA was isolated using TRIzol^®^ reagent as described by the manufacturer (Sigma, St. Louis, MO, USA) and treated with DNAse (Promega) to remove possible DNA contamination. One µg of total RNA was reverse transcribed using the Superscript III first-strand synthesis kit (Invitrogen, Waltham, MA, USA). RT-PCR primers are presented in Supplementary Table 2. PCR cycles were: initial denaturing cycle at 96 °C for 2 min, 30 cycles of 96 °C, 57 °C, and 72 °C for 30 s each; followed by a final extension cycle at 72 °C for 5 min. Twenty-five µL of PCR product was run on a 1% Tris-acetate-EDTA agarose gel for visualisation.

### Quantitative RT-PCR

qRT-PCR was used to compare *chmp7* mRNA levels between genotypes. RNA was extracted from *chmp7*^*+/+*^, *chmp7*^*+/−*^, and *chmp7*^*−/−*^ embryos at 6 dpf, and pooled with a constant number of fish (20–25) per genotype within each biological replicate. cDNA was prepared as above. qRT-PCR was performed using a Lightcycler 480 (Roche, Basel, Switzerland) and SYBR Green master mix (Roche). An average of actin, beta 1 (*actb1*), 18s ribosomal RNA (*18SrRNA*), and eukaryotic translation elongation factor 1 alpha 1 (*eef1α1*) expression values was used as a reference. Primers are presented in Supplementary Table 2. Three technical replicates were conducted for each biological replicate. Generalised mixed linear modelling was used to examine differences in mRNA levels. Genotype was examined as a fixed effect, with biological replicate as a random effect. F tests were performed using Satterthwaite estimated degrees of freedom.

### 24-h locomotion assay: 6 dpf

Zebrafish activity levels were examined using locomotion assays at 6 dpf to examine activity at a larval stage that demonstrated autonomous movement, while still utilising a high throughput behavioural assay. Embryos were collected between 09:00 and 10:00 and raised in Petri dishes in a 14-h day (09:00-23:00, 300 lux ± 20 lux) and 10-h night (23:00-09:00, full darkness) cycle until 6 dpf. Larvae were fed 0.5 ml concentrated paramecium between 09:00 and 10:00 on day 5 and 6, and medium was changed daily between 14:00 and 16:00. Between 14:00 and 16:00 on day 6, larvae were transferred to 24-well plates containing 1.5 ml of E3 embryo medium (5 mm NaCl, 0.17 mm KCl, 0.33 mm CaCl, 0.33 mm MgSO4 in water) per well to acclimatise. Between 22:30 and 22:50 on day 6, plates were transferred to Zebraboxes (Viewpoint, Lyon, France). At 22:50, Zebraboxes were closed to allow fish to habituate to the darkness for 10 min, and video recording began at 23:00. The experiment ran for 24 h and 30 min in full darkness, after which embryos were sacrificed and genotyped.

### 24-h locomotion assay: drug treatment at 6 dpf

To examine the effect of methylphenidate, locomotion assays were performed as described above. However, at 22:00 on day 6, 150 µL of dH_2_O (vehicle control) or 100 µM of Threo-methylphenidate hydrochloride (Tocris Bioscience, Bristol, United Kingdom) was added to wells containing 1.35 ml of E3 medium and the fish, to yield a final volume of 1.5 ml per well and 10 µM methylphenidate, as described by Lange et al.^20^. Raised lids were used to prevent evaporation, any wells showing condensation were removed from analysis. For each experiment, methylphenidate and vehicle were randomised across the plate, and the investigator blinded to treatment by a third party. Blinding was removed after initial mixed model tests.

### 24-h locomotion assay: 6- & 12-weeks post-fertilisation

Locomotion assays were used to examine activity levels in larval and adult fish. Fish were genotyped at 3 dpf by extracting DNA from fin clippings using 50 mM NaOH and 1 M Tris-HCl (pH 7.5), and then sorted by genotype. Fish were raised under a day-night cycle of 12 h day and 12 h night (08:00-20:00, and 20:00-08:00 respectively). Between 12:00 and 14:00 on day 41 and day 83, fish were transferred to individual tanks to acclimatise. Between 19:00 and 19:50 on day 41 and 83, tanks were transferred to Zebracubes (Viewpoint, 9 tanks per system). At 19:50, Zebracubes were closed to allow fish to habituate to the darkness for 10 min, and tracking began at 20:00. Positions of genotypes were randomised, and the investigator blinded to genotype. Video tracking ran for 24 h and 30 min in full darkness, after which fish were returned to their tanks.

### Video analysis

Videos were analysed using Ethovision (Noldus, version 14, Wageningen, Netherlands). Thresholds were: Moving, 1 mm/sec; Stopping, 0.75 mm/sec; Detection threshold, Dynamic Subtraction, Darker, 9. Voxel smoothing was used to remove errors in 6 dpf analyses, with movements < 0.04 mm and > 12 mm per frame excluded.

### Locomotion assay statistical analysis

Data were processed using Microsoft Excel 2013 (Microsoft, Redmond, WA, USA) and statistical analyses performed using SPSS Statistics 26 (IBM, Armonk, NY, USA). Data were ordered chronologically into 10-min bins. Time points at the end of videos less than 300 s long were excluded. For each fish, activity data were summed by hour. A normalised value for each fish per hour was determined by comparing activity, per fish per hour, to the average activity value of all fish per hour, from the respective replicate. Genotypes were then assigned to individual fish, and fish with ambiguous genotypes removed from analysis. Data points from the 30 min past the initial 24 h were excluded. Data were visualised using a line graph in GraphPad Prism Version 8 (GraphPad Software, San Diego, CA, USA).

To examine differences in activity, a mixed linear model was used. For the 6, 42, and 84 dpf locomotion assays, main effects of time and genotype, and an interaction effect of time by genotype were used. Repeated measures of time (h) were modelled using a first-order autoregressive variance structure for the 6 dpf assay, and a diagonal variance structure was used for the 42 dpf and 84 dpf assays. Random effects of Zebrabox tracking system, as well as individual animals grouped by genotype, were used. A natural log transformation was applied to the normalised data to meet assumptions of normality which were checked by inspection of the residuals. F tests were performed using a maximal likelihood model, with Satterthwaite estimated degrees of freedom. For the drug treatment assays main effects of time, treatment, and genotype, and an interaction effect of time by genotype by treatment were used. All pairwise comparisons for time points were two-tailed, performed using Bonferroni adjustment for multiple comparisons.

### Confocal microscopy live imaging

*chmp7*^*+/−*^ fish were crossed to a green fluorescent protein (GFP)-tagged HuC reporter (*HuC:eGFP*^21^) transgenic (Tg) line and raised to adulthood. *Tg(HuC:eGFP);chmp7*^*+/*−^ fish were crossed to *chmp7*^*+/*−^ fish, and embryos raised in E3 containing 200 µM N-Phenylthiourea (PTU, Sigma) from 6 h to suppress melanocyte formation, changing medium every 48 h. Embryos were sorted for fluorescence at 2 dpf. At 3 dpf, fish were anaesthetised using 0.0016% Tricaine methanesulfonate (Sigma) in E3, their tails clipped, DNA extracted, and fish sorted by genotype. In order to examine brain volume at a larval stage that was in line with the locomotion assays, at 6 dpf, embryos were again anesthetised and set in 1% low melting agarose in E3 containing tricaine in 0.8 mm fluorinated ethylene propylene (FEP) tubing (Bola, Grünsfeld, Germany). Genotypes were randomised, and the investigator blinded to genotype. Images were taken using a Thorlabs confocal microscope (Newton, NJ, USA), with an Olympus 20x water dipping 1.0 NA objective (Tokyo, Japan), pinhole 25 µm, 2.005 µm/pixel, step size = 1 µm, averaging = 16 frames.

### Brain image registration and analysis

Image registration of live confocal stacks was achieved using Advanced Normalization Tools (ANTs) registration software (3.0.0.0), running on Monash University’s MASSIVE computing cluster. Registered images were analysed using cobraZ brain volume analysis software as described by Gupta et al.^22^. In addition to total volume, brain regions homologous to human regions known to have volume differences in ADHD individuals^4^ (telencephalon (pallium, subpallium, anterior commissure), thalamus, and ventral thalamus) were examined using generalised mixed linear modelling. Genotype was examined as a fixed effect, with biological replicate as a random effect. F tests were performed with Satterthwaite estimated degrees of freedom. Bonferroni corrections for multiple comparisons were applied to each test.

## Results

### *chmp7* is expressed throughout early development

**Fig. 1:**
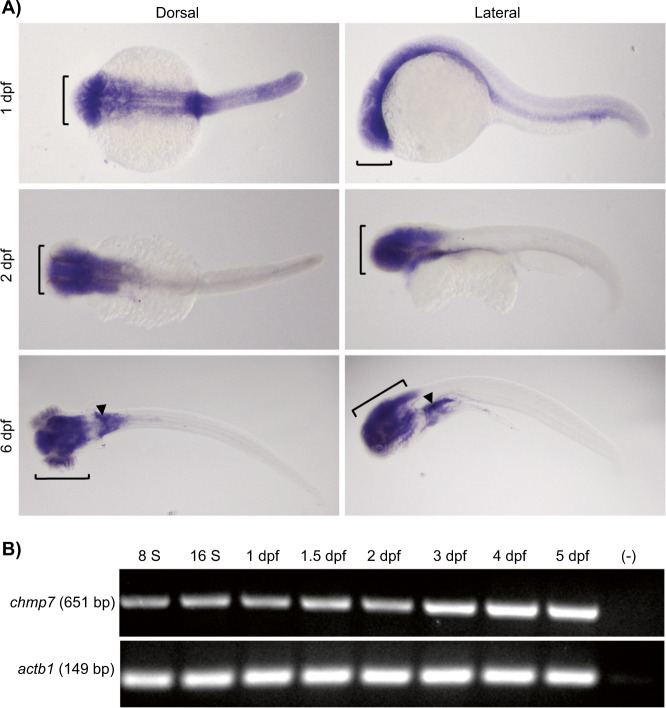
Characterisation of *chmp7* expression. **a** Whole-mount in situ hybridisation on zebrafish at 1, 2, and 6 dpf, using DIG-labelled RNA probes specific to zebrafish *chmp7*. [: head, ▼: kidney. **b** RT-PCR for *chmp7* during early zebrafish development. *actb1* was amplified as a positive control. – is the no template negative control.

Zebrafish possess orthologues of all members of the human CHMP family genes (Supplementary Fig. 2, Supplementary Table 3.). More importantly, the zebrafish Chmp7 protein has a sequence identity of 51% and similarity of 70% to that of human. To identify where and when *chmp7* is expressed in zebrafish, in situ hybridisations and RT-PCR were performed on wildtype embryos. We observed that *chmp7* was expressed ubiquitously in the embryo at 1 day post-fertilisation (dpf), with higher expression levels in the brain, becoming more restricted to the head by 2 dpf. It remained visible only in the head and kidney at 6 dpf (Fig. 1A). RT-PCR showed that *chmp7* was expressed at the 8-somite stage through to at least 5 dpf (Fig. 1B).

### *chmp7* heterozygotes have reduced mRNA levels

After confirming that *chmp7* expression was detectable during early zebrafish development, CRISPR-Cas9 genome editing was used to mutate the gene, resulting in a 7 bp deletion in exon 2 (Supplementary Fig. 3A). This results in the addition of 20 amino acids, and a stop codon following the 142nd amino acid (Supplementary Fig. 3B). This is predicted to remove the sucrose non-fermenting protein 7 (Snf7) domain, the main catalytic domain for CHMP7 responsible for its interaction with CHMP4B and thus, ESCRT-III^10^.

**Fig. 2:**
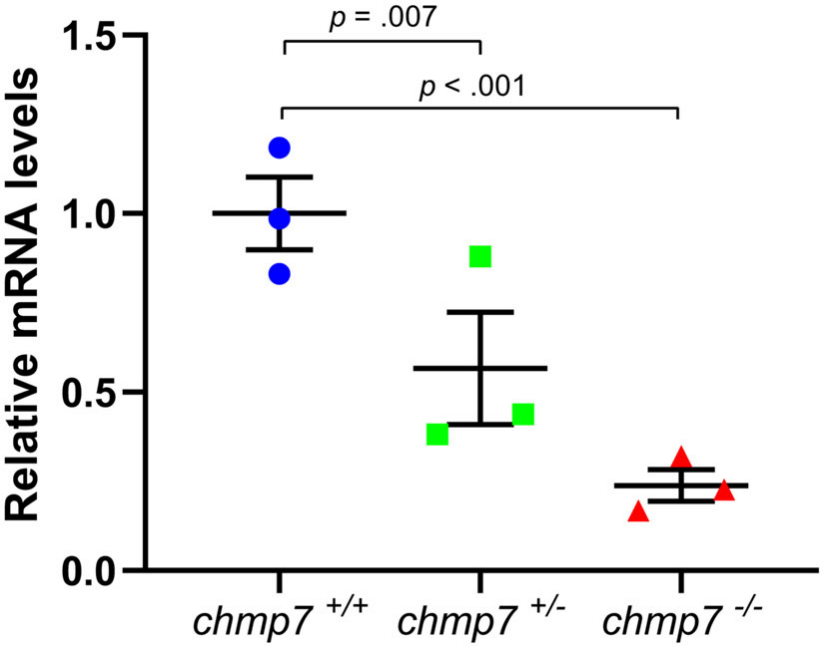
*chmp7* qRT-PCR on *chmp7*^+/+^, *chmp7*^+/−^, and *chmp7*^−/−^ embryos. Mixed linear modelling demonstrated a significant difference between genotypes (*p* = 0.001). Data are from three biological replicates, and is normalised to *chmp7*^*+/+*^ values. Centre lines = mean, error bars = ± standard error of the mean (SEM).

This mutation is predicted to trigger nonsense mediated decay, and a loss of protein, rather than the production of a truncated isoform. To determine if *chmp7*^*+/−*^ fish have reduced *chmp7* mRNA, thereby mimicking the reduction observed in individuals homozygous for the ADHD risk allele (T) of the ADHD-associated SNP, quantitative real-time PCR (qRT-PCR) on cDNA from *chmp7*^*+/+*^, *chmp7*^*+/−*^, and *chmp7*^*−/−*^ 6 dpf fish was performed. Mixed linear modelling demonstrated a significant reduction in mRNA levels (*F* = 71.41 (2, 4), *p* = 0.001, Fig. 2), and *chmp7*^*+/*−^ fish had 53% total *chmp7* mRNA compared to *chmp7*^*+/+*^ (*t* = −5.13 (4), *p* = 0.007), supporting the use of *chmp7*^*+/*−^ fish as a model of the reduction in *CHMP7* mRNA levels correlated with the rs2294123 homozygous ADHD risk allele (T).

### *chmp7* heterozygous larvae display a hyperactivity phenotype

Given that *chmp7*^*+/−*^ fish possess similar reductions in *chmp7* mRNA levels to individuals homozygous for the *CHMP7* ADHD risk allele, we examined if reduced *chmp7* mRNA levels lead to a hyperactivity phenotype at embryonic (6 dpf), juvenile (42 dpf), and adult (84 dpf) stages.

**Fig. 3:**
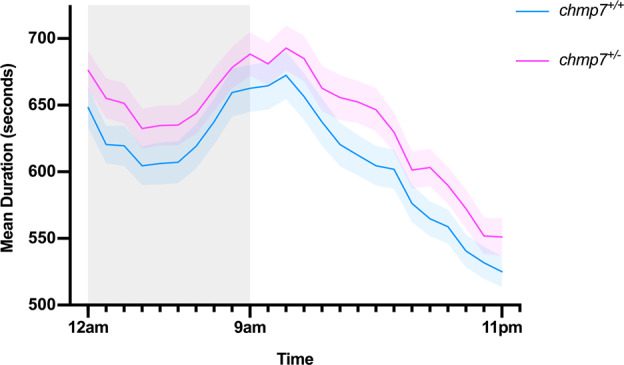
Activity analysis of *chmp7*^*+/+*^ (*n* = 153) and *chmp7*^*+/−*^ (*n* = 131) zebrafish 6 dpf embryos over a 24-h period. Average time spent moving in seconds each hour per genotype is displayed on the *Y*-axis. Data are from three biological replicates. Error bars = ± SEM.

The activity of *chmp7*^*+/+*^ (*n* = 153) and *chmp7*^*+/−*^ (*n* = 131) zebrafish at 6 dpf was tracked over a period of 24 h, starting at 6 days, 14 h post-fertilisation (hpf) (Fig. 3). Mixed linear modelling analysis demonstrated a significant main effect of genotype (*F* = 4.70 (1, 287.06), *p* = 0.031). There was no significant interaction effect of genotype and time (*F* = 0.77 (23, 3543.20), *p* = 0.78). This demonstrates that *chmp7*^*+/*−^ fish are consistently more active than *chmp7*^*+/+*^ fish over the 24-h period.

To determine if the hyperactivity phenotype persists into juvenile and adult stages, activity of *chmp7*^*+/+*^ and *chmp7*^*+/−*^ fish was tracked over a period of 24 h, starting at 41 days 11 hpf for juveniles, and 83 days 11 hpf for adults. There were no significant differences between genotypes over the entire experimental period for either juveniles (*chmp7*^*+/+*^, *n* = 41, *chmp7*^*+/−*^, *n* = 50, *F* = 0.29 (1, 61.52), *p* = 0.59, Supplementary Fig. 4A) or adults (*chmp7*^*+/+*^, *n* = 30, *chmp7*^*+/−*^, *n* = 36, *F* = 0.008 (1, 60.55), *p* = 0.93, Supplementary Fig. 4B), suggesting the hyperactivity phenotype does not persist past the larval stage.

### *chmp7* heterozygotes have significantly smaller brain volumes

**Fig. 4:**
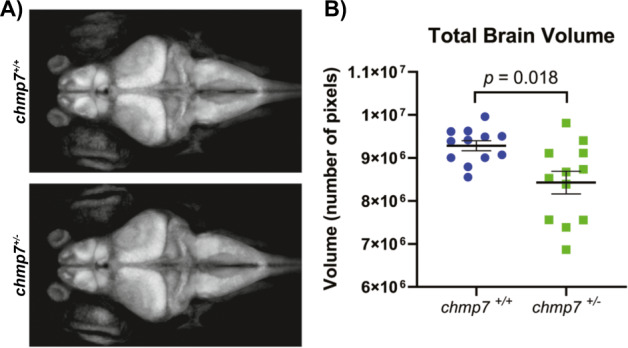
Total brain volume is decreased in *chmp7* heterozygotes at 6 dpf. **a** Maximum intensity projections of whole brains from *Tg(HuC:eGFP);chmp7*^*+/+*^ (*n* = 12) and *Tg(HuC:eGFP);chmp7*^*+/−*^ (*n* = 12) 6 dpf fish. **b** *chmp7*^*+/−*^ fish had significantly reduced total brain volumes when compared to *chmp7*^*+/+*^ fish. Data are from three biological replicates. Centre lines = mean, error bars = ± SEM.

Given the consistently reported reduced brain volumes for individuals with ADHD, the heads of *chmp7*^*+/+*^ (*n* = 12) and *chmp7*^*+/−*^ (*n* = 12) zebrafish on a pan-neuronal fluorescent *Tg(HuC:eGFP)* background, were imaged live using confocal microscopy at 6 dpf (Fig. 4A). We observed a 9.2% total brain volume reduction in *chmp7*^*+/−*^ fish compared to *chmp7*^*+/+*^ fish (*F* = 11.49 (1, 20), *p* = 0.018, one-tailed, Fig. 4B). However, we did not observe significant differences for specific selected brain regions (Supplementary Table 4).

### Methylphenidate significantly reduces hyperactivity in *chmp7* heterozygotes

**Fig. 5:**
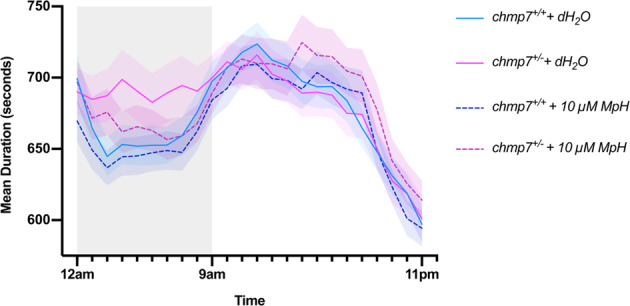
Activity analysis of *chmp7*^+/+^ and *chmp7*^+/−^ zebrafish 6 dpf embryos over a 24-h period, both treated with either 10 µM methylphenidate (MpH) or dH_2_O. Average time spent moving in seconds each hour per genotype is displayed on the *Y* axis. Data are from six biological replicates. Error bars = ± SEM.

To determine if methylphenidate could ameliorate the hyperactivity seen in *chmp7*^*+/−*^ fish, *chmp7*^*+/+*^ + dH_2_O (*n* = 179), *chmp7*^*+/−*^ + dH_2_O (*n* = 160), *chmp7*^*+/+*^ + 10 µM methylphenidate (*n* = 171), *chmp7*^*+/−*^ + 10 µM methylphenidate (*n* = 166) zebrafish were tracked over a period of 24 h starting at 6 days, 14 hpf. *chmp7*^*+/−*^ + dH_2_O fish demonstrated hyperactivity compared to *chmp7*^*+/+*^ + dH_2_O fish during the 10-h night period (Fig. 5). However, this effect was diminished in the *chmp7*^*+/−*^ + methylphenidate fish. Mixed linear modelling demonstrated a significant interaction between genotype, drug treatment, and time (*F* = 1.60 (69, 8038.37), *p* = 0.001). Given the significant interaction of genotype and treatment over time, we investigated the differences between groups across time.

*chmp7*^*+/−*^ + dH_2_O fish demonstrated significant hyperactivity compared to *chmp7*^*+/+*^ + dH_2_O fish across the majority of the night period (hour 3, *p* = 0.002; hour 4, *p* = 0.007; hour 5, *p* = 0.013; hour 6, *p* = 0.020; hour 7, *p* = 0.025; hour 8, *p* = 0.014). Application of methylphenidate gradually reduced the activity of *chmp7*^*+/−*^ + methylphenidate fish until it was significantly less than *chmp7*^*+/−*^ + dH_2_O fish (hour 8, *p* = 0.045). In addition, *chmp7*^*+/−*^ + methylphenidate fish were not significantly different from *chmp7*^*+/+*^ + dH_2_O fish for the majority of the night period. This demonstrates that the application of methylphenidate was sufficient to significantly reduce the hyperactivity seen in *chmp7*^*+/−*^ fish to levels comparable to that of wildtype.

## Discussion

Zebrafish are emerging as a promising model for neuropsychiatric disorders^23^. We demonstrate here for the first time the utility and versatility of zebrafish models to validate ADHD genetic associations through analysis of swimming activity and brain volume as phenotypes of ADHD. The hyperactivity observed in 6 dpf *chmp7*^*+/−*^ fish did not persist into adulthood, demonstrating the use of zebrafish for testing the temporal progression of ADHD phenotypes. Zebrafish can also be used to examine changes in brain volume commonly observed in ADHD individuals^4^, providing anatomical evidence for ADHD-associations. Finally, the response to methylphenidate suggests that dysregulation of dopamine (or noradrenaline) signalling could be contributing to the observed hyperactivity. In addition, given the effectiveness of methylphenidate varies between individuals with ADHD^24^, the use of zebrafish for testing ADHD-associated gene models for their response to drugs may be beneficial for understanding drug response variability.

The molecular mechanism behind the hyperactivity of *chmp7*^+/−^ fish requires further investigation. Given the positive response of *chmp7*^*+/−*^ fish to methylphenidate treatment, this is suggestive of abnormalities in either dopamine or noradrenaline signalling. While it is possible that other neurotransmitter pathways could be contributing to the phenotype we see in *chmp7*^*+/−*^ fish, abnormal dopaminergic signalling, in particular, has been repeatedly associated with ADHD^25^. As such, investigating the role of *CHMP7* in dopamine signalling requires further investigation. Additionally, defects in neuronal pruning are seen in knockdown^26^, loss of function^27^, and dominant negative mutations^28^, of ESCRT-III proteins. Given CHMP7’s known interactions with ESCRT-III proteins, it is possible that neuronal pruning is also disrupted when *CHMP7* mRNA levels are reduced. The hyperactivity phenotype of *chmp7*^*+/−*^ fish could therefore be due to delayed maturation of neural networks important for impulse control, or motor control/coordination, due to a lack of efficient neural pruning. This would be consistent with the neurodevelopmental delay seen in ADHD individuals (reviewed in Dark et al.^29^).

The assays presented in Figs. 3 and 5 identified significant hyperactivity in *chmp7*^*+/−*^ fish compared to *chmp7*^*+/+*^. However, for the *chmp7*^*+/−*^ + dH_2_O control in the methylphenidate assay which essentially replicated the previous activity experiment, the hyperactivity was restricted to the night period. This could indicate that a reduction in *chmp7* mRNA has stronger, more consistent impacts on sleep patterns rather than waking cognition. This is consistent with sleep impairments often seen in ADHD, as well as inter- and intra-subject variability in circadian rhythms^30^. In addition, hyperactivity during night periods was observed in pan-neuronal knockdown of the dopamine transporter, or *latrophilin*, in *Drosophila*, which was suggested to be characteristic of dysregulation of dopamine signalling^31^. This highlights that dopamine imbalance could be contributing to the hyperactivity phenotype observed in *chmp7*^*+/−*^ fish. This is consistent with the amelioration of the phenotype by methylphenidate application (Fig. 5).

This study is the first of its kind to functionally examine *CHMP7* as a risk gene for ADHD using an animal model. It is also the first functional validation using zebrafish of an ADHD-associated gene identified through GWAS. We demonstrate here that *chmp7*^*+/−*^ fish are an appropriate model for the *CHMP7* ADHD-associated alleles, and that the decrease in *chmp7* mRNA levels is correlated with a hyperactivity phenotype and reduced brain volume. Furthermore, this hyperactivity is alleviated by treatment with methylphenidate, contributing to our understanding of specific ADHD risk factors to variability in drug responsiveness. More generally, we advocate for the use of zebrafish to model the large number of candidate genes for ADHD that have recently emerged from large-scale GWAS^8^.

## Supplementary information

Supplemental Material

## Data Availability

Raw data and statistical analysis files are available at https://bridges.monash.edu/projects/Functional_validation_of_CHMP7_as_an_ADHD_risk_gene/83780.
