# Supplementary material for: Functional validation of CHMP7 as an ADHD risk gene
Source: Transl Psychiatry. 2020 Nov 6;10:385. doi: 10.1038/s41398-020-01077-w (PMC7648633; doi:10.1038/s41398-020-01077-w)
Supplement: Supplementary file 1 — Supplemental Material [file 41398_2020_1077_MOESM1_ESM.docx]

**Supplementary Material for:**

**Functional validation of *CHMP7* as an ADHD risk gene**

Callum Dark^1^, Caitlin Williams^1^, Mark A. Bellgrove^2^, Ziarih Hawi^2^, and Robert J. Bryson-Richardson^1^

^1^ School of Biological Sciences, Faculty of Science, Monash University

^2^ Turner Institute for Brain and Mental Health, School of Psychological Sciences, Monash University


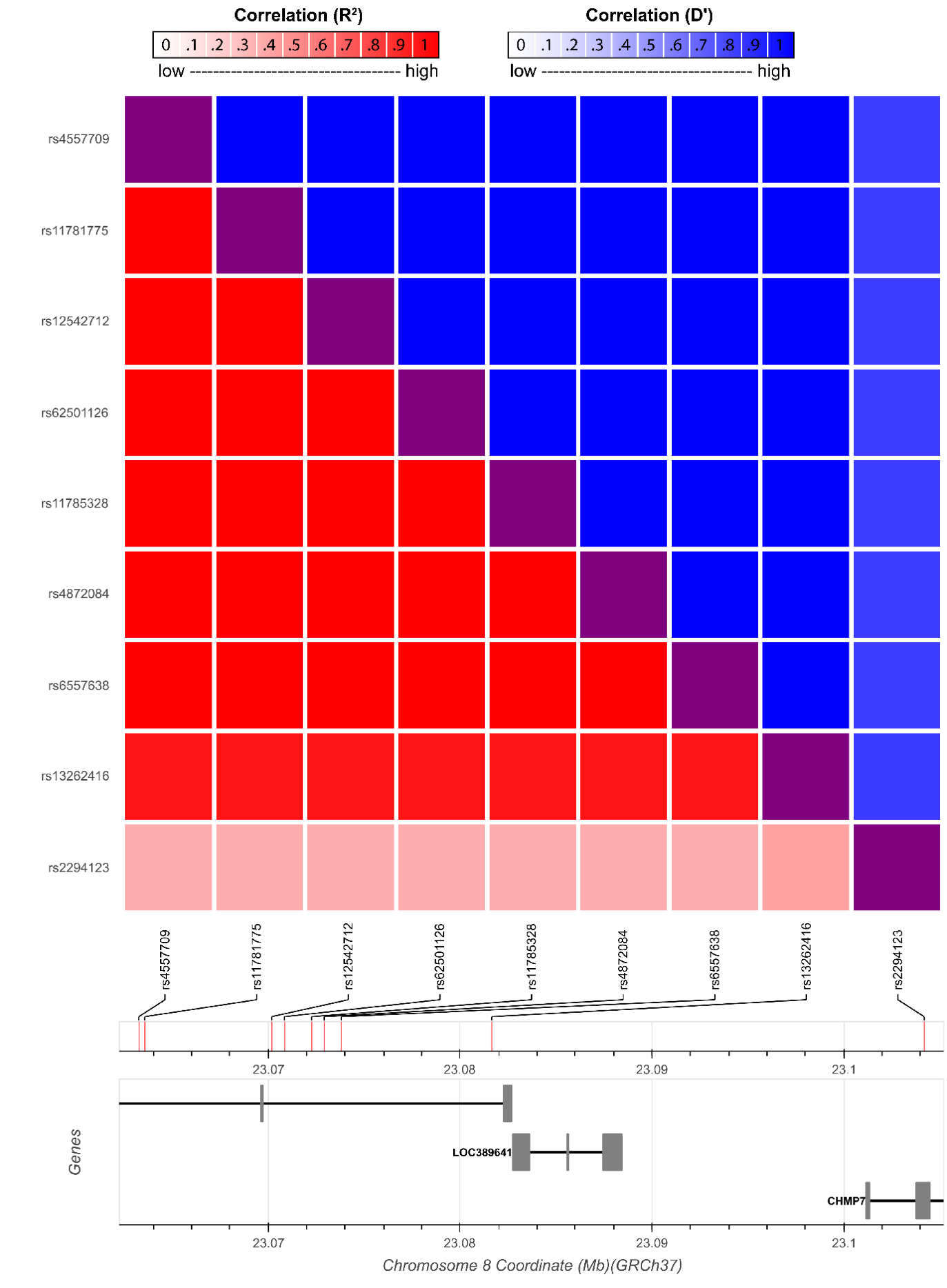


**Supplementary Figure 1**. Linkage disequilbrum (LD) relations between rs2294123 (mapped to CHMP7) and SNPs showing evidence of association in the meta-analysis of the Psychiatric Genetic Consortium GWAS^1^. LD relations were calculated from the Caucasian British population using LDlink; a bioinformatic tool designed to investigate patterns of LD across a variety of ancestral population groups^2^. The level of relationship between two SNPs for R^2^ and Dˈ is indicated by a colour gradient shown in the scales above.


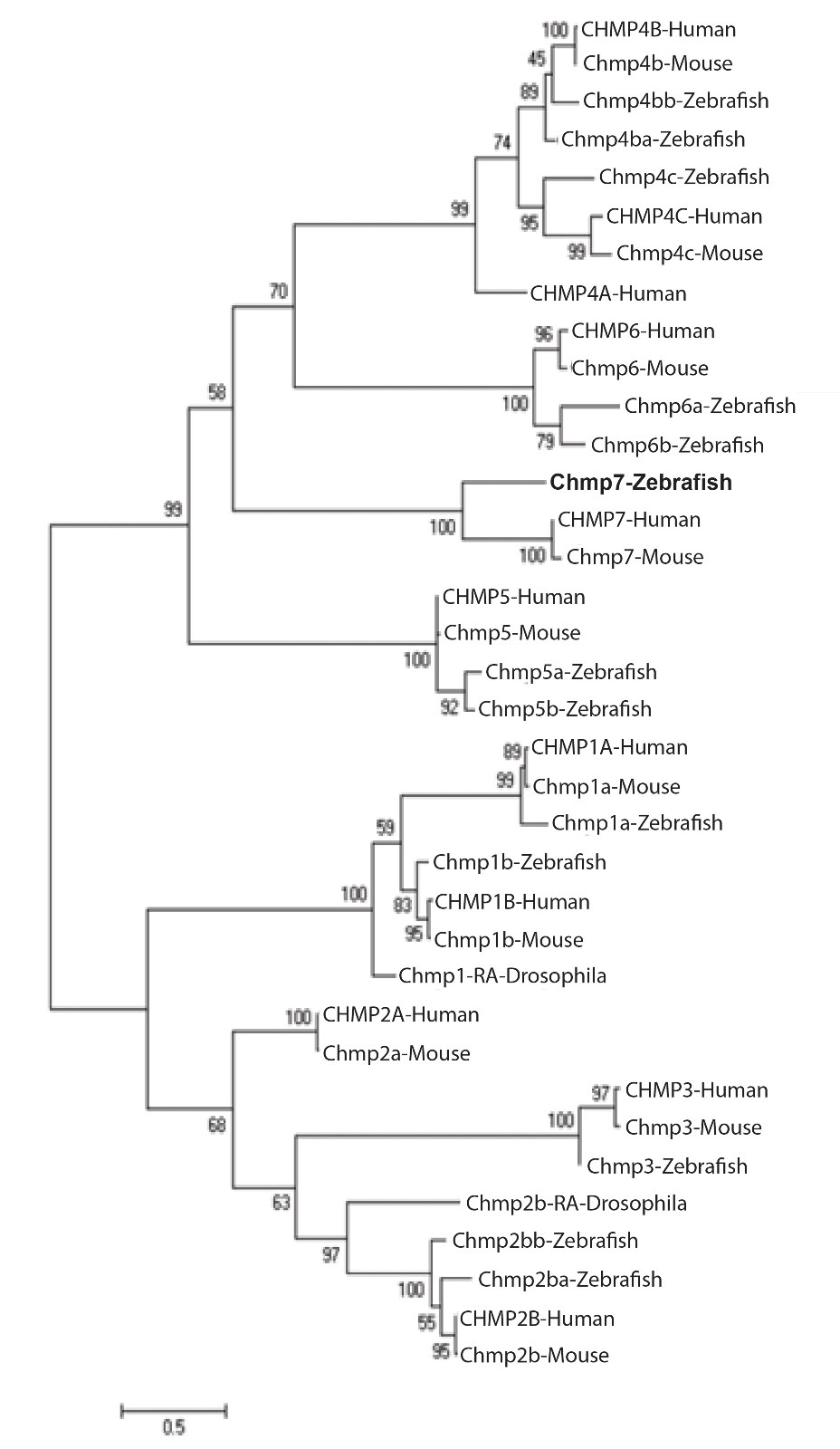


**Supplementary Figure 2.** Phylogenetic tree of the CHMP family in humans, mice, *Drosophila*, and zebrafish. All seven members of the CHMP family known in humans and mice are also identified in zebrafish. CHMP protein sequences from human, mouse, zebrafish, and *Drosophila* were aligned using ClustalX (Version 2.1^3^, Supplementary Table 3). Evolutionary history was inferred using the Maximum Likelihood method based on the Le Gascuel 2008 model^4^. The percentage of trees in which the associated taxa clustered is shown next to the branches. Initial tree(s) for the heuristic search were obtained by applying the Neighbor-Joining method to a matrix of pairwise distances estimated using a Jones-Taylor-Thornton (JTT) model^5^. A discrete Gamma distribution was used to model evolutionary rate differences among sites (5 categories (+G, parameter = 5.0837)). The tree was calculated from the alignment using Molecular Evolutionary Genetics Analysis (MEGA) software version 6^6^, and bootstrap values taken from 1000 repetitions using the Le Gascuel 2008 model.


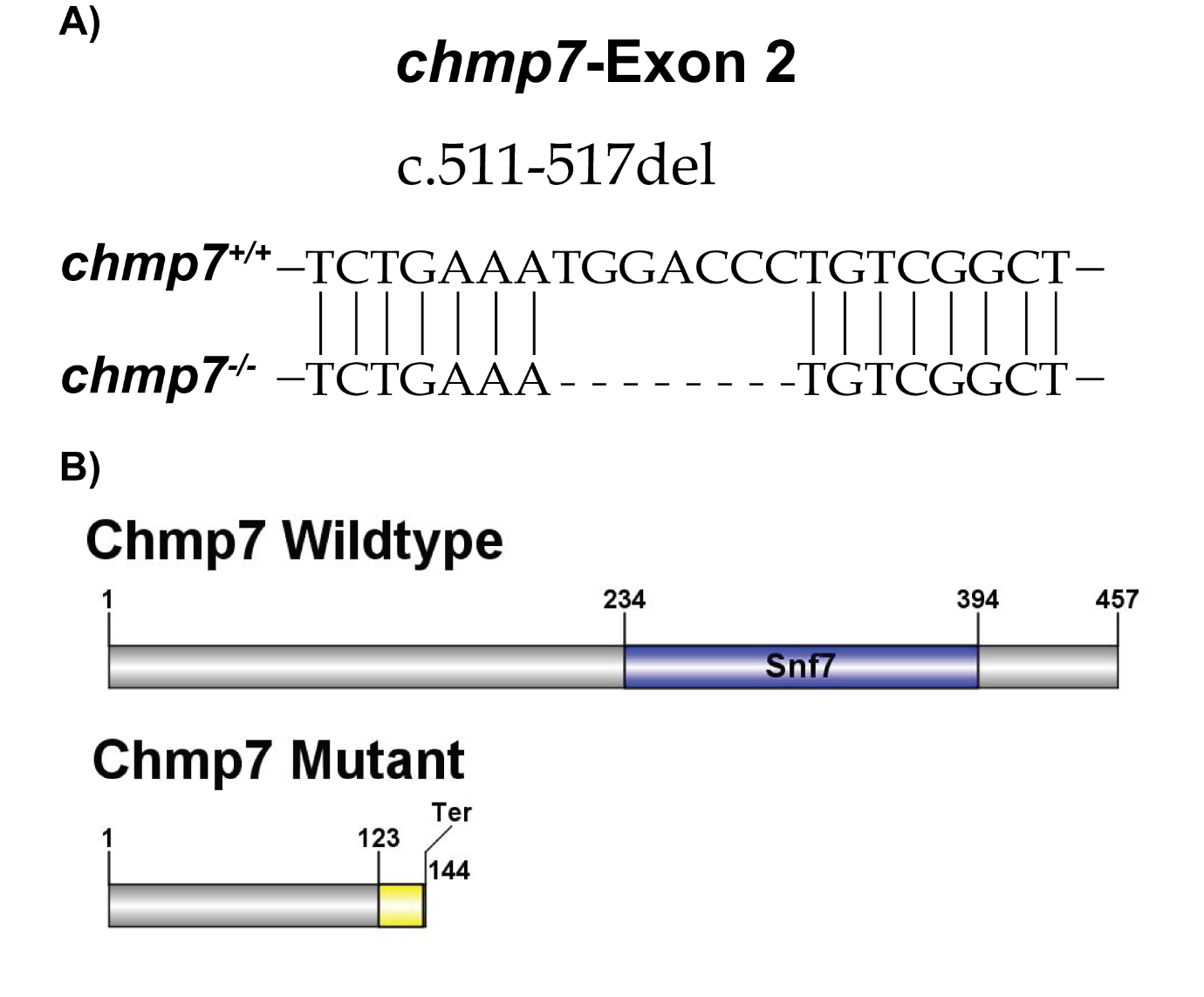


**Supplementary Figure 3.** **A)** CRISPR-Cas9 genome editing was used to induce a mutation in *chmp7*, resulting in a 7 bp deletion at positions 511-517 in exon 2. **B)** Schematics of the Chmp7 wildtype and mutant (p.(Trp124CysfsTer20)) proteins. Insertion of 20 amino acids at position 123 is followed by the addition of a premature STOP codon (Ter). This is predicted to result in the complete removal of the Snf7 domain from the Chmp7 mutant protein.


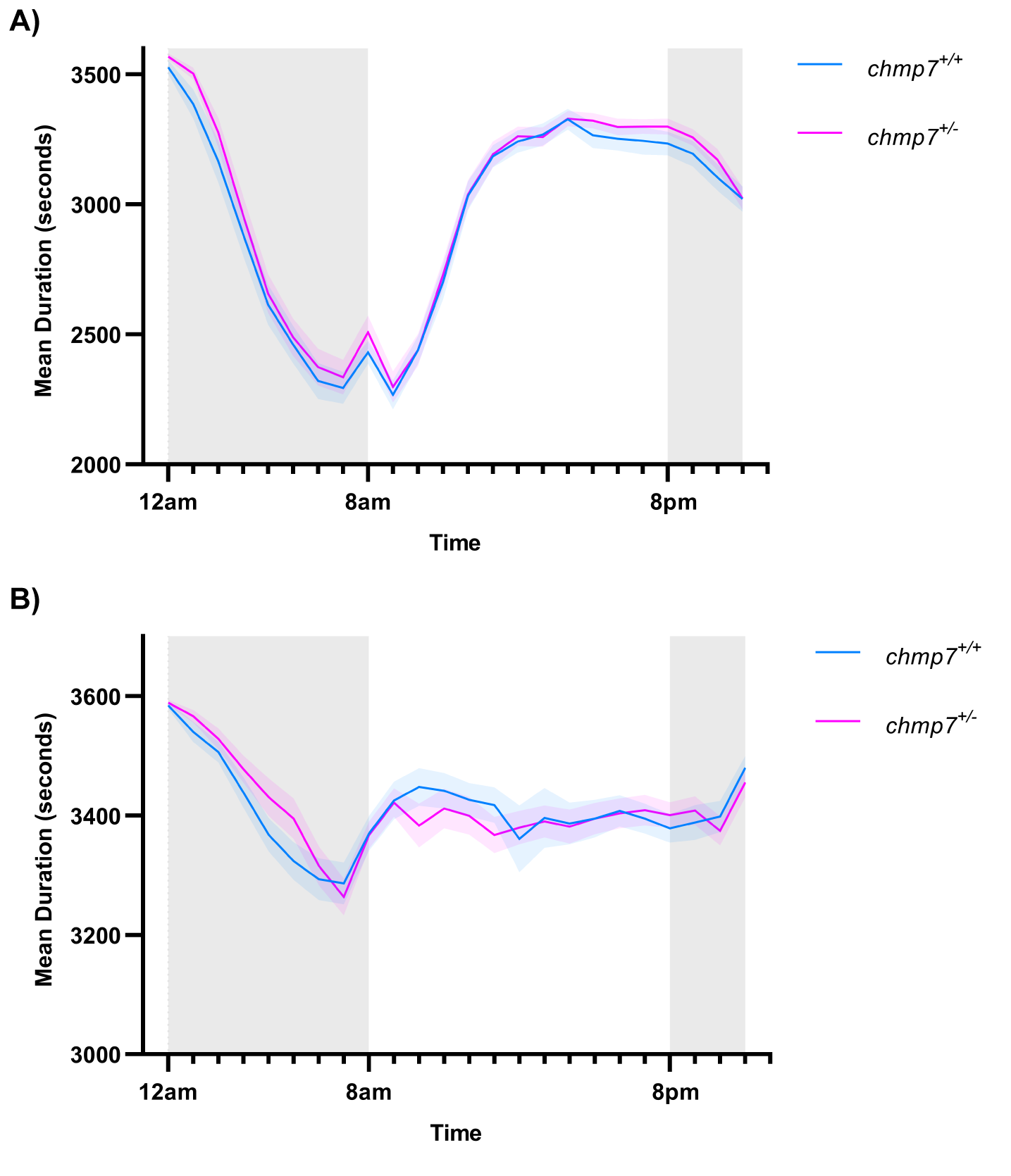


**Supplementary Figure 4.** Activity analysis of *chmp7^+/+^* and *chmp7^+/-^* zebrafish at **(A)** 42 dpf (*chmp7^+/+^*, *n* = 41; *chmp7^+/-^*, *n* = 50) and **(B)** 84 dpf (*chmp7^+/+^*, *n* = 30; *chmp7^+/-^*, *n* = 36) over a period of 24 hours. No significant differences were seen between genotypes at both time points. The average time in seconds spent per genotype moving per hour time point is displayed on the Y axis. Data is from five biological replicates for 6 weeks, and 4 replicates for 12 weeks. Error bars = +/- SEM.

| **Supplementary Table 1.** Linkage disequilibrium (LD) relationships (presented as Dˈ and R^2^) between rs2294123 and several SNPs (closely mapped to CHMP7) showing evidence of association with ADHD in a large meta-analysis of ADHD-GWAS^1^. | | | | | | | | | | | |
| --- | --- | --- | --- | --- | --- | --- | --- | --- | --- | --- | --- |
| **D' LD** | | | | | | | | | | | |
| **RS number** | **rs4557709** | **rs11781775** | **rs12542712** | **rs62501126** | **rs11785328** | **rs4872084** | **rs6557638** | **rs13262416** | **rs2294123** | **Demontis et al.,**^1^ **-P value** | **Distance to rs2294123** |
| **rs4557709** | 1 | 1 | 1 | 1 | 1 | 1 | 1 | 1 | 0.77 | 0.0067 | 40.93 Kp |
| **rs11781775** | 1 | 1 | 1 | 1 | 1 | 1 | 1 | 1 | 0.77 | 0.0061 | 40.63 Kb |
| **rs12542712** | 1 | 1 | 1 | 1 | 1 | 1 | 1 | 1 | 0.77 | 0.0085 | 34 Kb |
| **rs62501126** | 1 | 1 | 1 | 1 | 1 | 1 | 1 | 1 | 0.77 | 0.0088 | 33.4 Kb |
| **rs11785328** | 1 | 1 | 1 | 1 | 1 | 1 | 1 | 1 | 0.77 | 0.0087 | 31.9 Kb |
| **rs4872084** | 1 | 1 | 1 | 1 | 1 | 1 | 1 | 1 | 0.77 | 0.0096 | 31.3 Kb |
| **rs6557638** | 1 | 1 | 1 | 1 | 1 | 1 | 1 | 1 | 0.77 | 0.0079 | 30.4 Kb |
| **rs13262416** | 1 | 1 | 1 | 1 | 1 | 1 | 1 | 1 | 0.78 | 0.028 | 22.6 Kb |
| **rs2294123** | 0.77 | 0.77 | 0.77 | 0.77 | 0.77 | 0.77 | 0.77 | 0.78 | 1 | 0.3574 |  |
| **R^2^ LD** | | | | | | | | | | | |
| **RS number** | **rs4557709** | **rs11781775** | **rs12542712** | **rs62501126** | **rs11785328** | **rs4872084** | **rs6557638** | **rs13262416** | **rs2294123** |  |  |
| **rs4557709** | 1 | 1 | 1 | 1 | 1 | 1 | 1 | 0.92 | 0.33 | 0.0067 | 40.93 Kb |
| **rs11781775** | 1 | 1 | 1 | 1 | 1 | 1 | 1 | 0.92 | 0.33 | 0.0061 | 40.63 Kb |
| **rs12542712** | 1 | 1 | 1 | 1 | 1 | 1 | 1 | 0.92 | 0.33 | 0.0085 | 34 Kb |
| **rs62501126** | 1 | 1 | 1 | 1 | 1 | 1 | 1 | 0.92 | 0.33 | 0.0088 | 33.4 Kb |
| **rs11785328** | 1 | 1 | 1 | 1 | 1 | 1 | 1 | 0.92 | 0.33 | 0.0087 | 31.9 Kb |
| **rs4872084** | 1 | 1 | 1 | 1 | 1 | 1 | 1 | 0.92 | 0.33 | 0.0096 | 31.3 Kb |
| **rs6557638** | 1 | 1 | 1 | 1 | 1 | 1 | 1 | 0.92 | 0.33 | 0.0079 | 30.4 Kb |
| **rs13262416** | 0.92 | 0.92 | 0.92 | 0.92 | 0.92 | 0.92 | 0.92 | 1 | 0.37 | 0.028 | 22.6 Kb |
| **rs2294123** | 0.33 | 0.33 | 0.33 | 0.33 | 0.33 | 0.33 | 0.33 | 0.37 | 1 | 0.3574 |  |

| **Supplementary Table 2.** Primers used for generation and screening of the *chmp7* mutant line, generation of the *chmp7* *in situ* probe, RT-PCR and qRT-PCR experiments. | |
| --- | --- |
| **Primer** | **Sequence** |
| *chmp7* exon 2 gRNA sequence | GCCTCTGAAATGGACCCTGT |
| *chmp7* exon 2 STOP cassette | CCGGCCTCTGAAATGGACCCGTCATGGCGTTTAAACCTTAATTAAGCTGTTGTAGTGTCGGCTCTGCTGGGCAGT |
| *chmp7* exon 2 gRNA genotyping forward | TGTGGATTGAGCGTGTTTTC |
| *chmp7* exon 2 gRNA genotyping reverse | GGGCGAACAATTTTGACTTC |
| *chmp7* *in situ* template forward | GGACTTCATCCTGCTGCTTC |
| *chmp7* *in situ* template reverse | TGTCGCACAGCTCCTGTATC |
| pGEM-T Easy M13 Forward | TGTAAAACGACGGCCAGT |
| pGEM-T Easy M13 Reverse | CAGGAAACAGCTATGACCATG |
| *chmp7* RT/qRT forward | GTGCGACACTCAGGATGAAG |
| *chmp7* RT/qRT reverse | TAATGGGGTGTGTCGGGACT |
| *actb1* RT/qRT forward | GCATTGCTGACCGTATGCAG |
| *actb1* RT/qRT reverse | GATCCACATCTGCTGGAAGGTGG |
| *18srRNA* qRT forward | TCGCTAGTTGGCATCGTTTATG |
| *18srRNA* qRT reverse | CGGAGGTTCGAAGACGATCA |
| *eef1α1* qRT forward | CTGGAGGCCAGCTCAAACAT |
| *eef1α1* qRT reverse | ATCAAGAAGAGTAGTACCGCTAGCATTAC |

| **Supplementary Table 3**. Genes and sequences used for CHMP phylogenetic analysis | | |
| --- | --- | --- |
| **Organism** | **Gene** | **Sequence** |
| Human | CHMP1A | ENSP00000380998.3 |
|  | CHMP1B | ENSP00000432279.1 |
|  | CHMP2A | ENSP00000310440.1 |
|  | CHMP2B | ENSP00000263780.4 |
|  | CHMP3 | ENSP00000263856.4 |
|  | CHMP4A | ENSP00000324205.9 |
|  | CHMP4B | ENSP00000217402.2 |
|  | CHMP4C | ENSP00000297265.4 |
|  | CHMP5 | ENSP00000223500.7 |
|  | CHMP6 | ENSP00000317468.5 |
|  | CHMP7 | ENSP00000324491.7 |
| Mouse | CHMP1A | ENSMUSP00000000759.8 |
|  | CHMP1B | ENSMUSP00000147285.1 |
|  | CHMP2A | ENSMUSP00000005711.4 |
|  | CHMP2B | ENSMUSP00000004965.6 |
|  | CHMP3 | ENSMUSP00000109815.3 |
|  | CHMP4B | ENSMUSP00000036206.9 |
|  | CHMP4C | ENSMUSP00000029049.5 |
|  | CHMP5 | ENSMUSP00000030128.5 |
|  | CHMP6 | ENSMUSP00000026434.6 |
|  | CHMP7 | ENSMUSP00000047700.8 |
| Zebrafish | Chmp1a | ENSDARP00000141533.1 |
|  | Chmp1b | ENSDARP00000141620.1 |
|  | Chmp2ba | ENSDARP00000055865.6 |
|  | Chmp2bb | ENSDARP00000008354.7 |
|  | Chmp3 | ENSDARP00000055486.5 |
|  | Chmp4ba | ENSDARP00000017897.7 |
|  | Chmp4bb | ENSDARP00000023938.6 |
|  | Chmp4c | ENSDARP00000014221.6 |
|  | Chmp5a | ENSDARP00000115597.2 |
|  | Chmp5b | ENSDARP00000138817.1 |
|  | Chmp6a | ENSDARP00000127696.1 |
|  | Chmp6b | ENSDARP00000130680.1 |
|  | Chmp7 | ENSDARP00000060627.4 |
| *Drosophila* | Chmp1 | FBpp0074859 |
|  | Chmp2b | FBpp0076869 |

| **Supplementary Table 4.** Volumes of brain regions in *chmp7^+/+^* and *chmp7^+/-^* fish. Values are in number of pixels, with SEM in brackets. *P* values are corrected for multiple comparisons using the Bonferroni method. Individual regions can be visualised at http://vis.arc.vt.edu/projects/zbb/ ^7^. | | | |
| --- | --- | --- | --- |
| **Region** | **Average: *chmp7*^+/+^** | **Average: *chmp7^+^*^/-^** | ***p* value**  **(one-tailed)** |
| Total Brain Volume | 9286411.42 (115960.56) | 8427591.92 (261207.92) | 0.018 |
| Pallium (r005) | 100701 (2137.95) | 95892.92 (3019.91) | 1.00 |
| Pallium (r037) | 76365.92 (1424.93) | 71808.42 (2394.97) | 1.00 |
| Pallium (r076) | 99504.58 (2134.86) | 93779.08 (3345.13) | 1.00 |
| Pallium (r133) | 36432.92 (620.33) | 33704.08 (1138.39) | 0.48 |
| Pallium (r180) | 65179.33 (1472) | 61414.67 (2163.25) | 1.00 |
| Subpallium (r026) | 79100.83 (1589.65) | 76443 (2368.08) | 1.00 |
| Subpallium (r075) | 67461.67 (1295.72) | 63329.34 (2137.78) | 1.00 |
| Ventral Thalamus (r048) | 84930.17 (1403.96) | 80069.08 (2746.55) | 1.00 |
| Ventral Thalamus (r129) | 63853.33 (1123.07) | 58089.42 (1978.8) | 0.18 |
| Thalamus (r138) | 58817.75 (952.45) | 53701.08 (1880.9) | 0.24 |
| Anterior commissure (r137) | 80268.67 (1808.83) | 77571.42 (2258.61) | 1.00 |

**References**

1 Demontis D. *et al.* Discovery of the first genome-wide significant risk loci for attention deficit/hyperactivity disorder. *Nat Genet*. **51**, 63-75 (2019).

2 Machiela M.J. & Chanock S.J. LDlink: a web-based application for exploring population-specific haplotype structure and linking correlated alleles of possible functional variants. *Bioinforma Oxf Engl*. **31**, 3555–3557 (2015).

3 Larkin M.A. *et al.* Clustal W and Clustal X version 2.0. *Bioinforma Oxf Engl*. **23**, 2947–2948 (2007).

4 Le S.Q. & Gascuel O. An Improved General Amino Acid Replacement Matrix. *Mol Biol Evol*. **25**, 1307–1320 (2008).

5 Jones D.T., Taylor W.R. & Thornton J.M. The rapid generation of mutation data matrices from protein sequences. *Bioinformatics*. **8**, 275–282 (1992).

6 Tamura K., Stecher G., Peterson D., Filipski A. & Kumar S. MEGA6: Molecular Evolutionary Genetics Analysis Version 6.0. *Mol Biol Evol*. **30**, 2725–2729 (2013).

7 Tabor K.M. *et al.* Brain-wide cellular resolution imaging of Cre transgenic zebrafish lines for functional circuit-mapping. *Elife*. https://doi.org/10.7554/eLife.42687.001 (2019).
